# Recovery of microbial DNA by agar-containing solution from extremely low-biomass specimens including skin

**DOI:** 10.1038/s41598-023-46890-7

**Published:** 2023-11-11

**Authors:** Rina Kurokawa, Hiroaki Masuoka, Lena Takayasu, Yuya Kiguchi, Yusuke Ogata, Ryoko Miura-Kawatsu, Masahira Hattori, Wataru Suda

**Affiliations:** 1 Laboratory for Microbiome Sciences, RIKEN Center for Integrative Medical Sciences, 1-7-22 Suehiro-Cho Tsurumi-Ku, Yokohama, Kanagawa 230-0045 Japan; 2 Graduate School of Advanced Science and Engineering, Cooperative Major in Advanced Health Science, Waseda University, 3-4-1 Ohkubo Shinjuku-Ku, Tokyo, 169-8555 Japan; 3 Department of Human Ecology, The University of Tokyo, Tokyo, Japan; 4 Division of Research and Development, Biogenomics, Co., Ltd, Nagasaki, Japan; 5 Nancy E. and Peter C. Meinig School of Biomedical Engineering, Cornell University, Ithaca, NY USA; 6 Department of Computational Biology and Medical Sciences, Graduate School of Frontier Sciences, The University of Tokyo, Chiba, Japan

**Keywords:** Metagenomics, Microbiome

## Abstract

Recovering a sufficient amount of microbial DNA from extremely low-biomass specimens, such as human skin, to investigate the community structure of the microbiome remains challenging. We developed a sampling solution containing agar to increase the abundance of recovered microbial DNA. Quantitative PCR targeting the 16S rRNA gene revealed a significant increase in the amount of microbial DNA recovered from the developed sampling solution compared with conventional solutions from extremely low-biomass skin sites such as the volar forearm and antecubital fossa. In addition, we confirmed that the developed sampling solution reduces the contamination rate of probable non-skin microbes compared to the conventional solutions, indicating that the enhanced recovery of microbial DNA was accompanied by a reduced relative abundance of contaminating microbes in the 16S rRNA gene amplicon sequencing data. In addition, agar was added to each step of the DNA extraction process, which improved the DNA extraction efficiency as a co-precipitant. Enzymatic lysis with agar yielded more microbial DNA than conventional kits, indicating that this method is effective for analyzing microbiomes of low-biomass specimens.

## Introduction

The human skin, intestinal tract, oral cavity, vagina, and lungs interact with external factors and harbor microbes that constitute the microbiome^1^. Studies of the human microbiome during the past decade have progressed considerably thanks to the development of high-throughput sequencing workflows. Host–microbiome interactions have been implicated in age^2,3^, sex^4^, lifestyle^5^, diet^6^, and some diseases^7^, with a focus on the gut microbiome.

The human skin microbiome can also play important roles in cutaneous immune systems and may be useful for preventing and treating some skin diseases^8,9^. However, analyzing the human skin microbiome using high-throughput sequencing-based metagenomics remains technologically challenging due to the extremely low DNA content of the skin microbiome^10^. Moreover, environmental DNA is ubiquitous in kits and laboratory reagents commonly used for DNA extraction^11–14^. Microbial DNA derived from commercially available DNA extraction kits, reagents, or experimental environments can be unintentionally sequenced and critically affect the interpretation of results^15,16^.

To address these challenges, we developed a sampling solution containing agar, which significantly increased the amount of recovered microbial DNA from several skin sites compared with conventional sampling solutions. Analysis of 1,000-fold diluted saliva with very low microbial DNA showed that agar reduced the loss of DNA during the entire process of DNA extraction. We further employed agar as a reagent for extracting community DNA from extremely low-biomass specimens.

## Results

### Comparison of DNA recovery from skin sites using three sampling solutions

**Figure 1:**
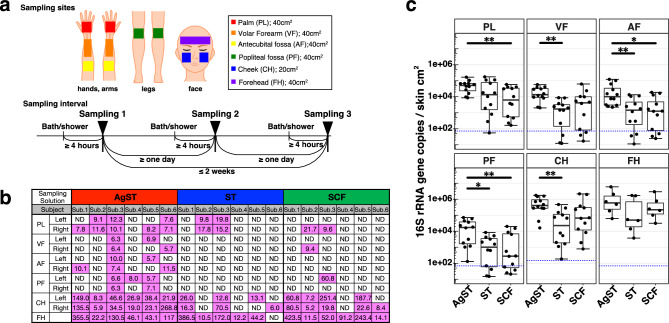
Skin sites and quantitation of DNA recovered using three sampling solutions. (**a**) Upper: Skin microbiome samples were collected from the forehead (FH) and the left and right sides of the palm (PL), volar forearm (VF), antecubital fossa (AF), popliteal fossa (PF), and cheek (CH) of six healthy individuals. Lower: Sampling intervals are shown. Skin sampling at certain skin sites was performed once a day with one solution. Skin sampling with another solution was performed on another day. The order of the three solutions, AgST, ST, and SCF, was randomized. (**b**) The amount (ng) of whole DNA per whole 50 µL DNA solution (pink) extracted from skin samples in three solutions was measured using Qubit HS. ND, not detectable: below the detection limit of the Qubit HS assay. (**c**) Boxplots show comparisons of 16S rRNA gene copies in skin from six body parts in three solutions. Vertical axis: number of 16S rRNA gene copies per cm^2^ of skin. * *P* < 0.05 and ** *P* < 0.01 (Wilcoxon signed-rank test). Broken blue lines: lower limit of qPCR standard curve. The values for all negative controls are below the broken blue line.

We obtained 198 specimens from 11 skin sites in six individuals using swabs with the following sampling solutions: ST^17–19^, SCF^20^, and ST containing 0.2% (w/v) agar (AgST) (Supplementary Table S1). We swabbed the forehead (FH) and the left and right sides of the palm (PL), volar forearm (VF), antecubital fossa (AF), popliteal fossa (PF), and cheek (CH) (Fig. 1a).

All skin specimens were placed in the sampling solutions and were immediately stored at −80 °C. Then, whole DNA was extracted using enzymatic lysis^21^ and photochemically quantified using Qubit (see Methods). AgST had more samples with DNA content above the Qubit detection limit than ST (*P* = 1.78e-05, Fisher’s exact test) and SCF (*P* = 0.0042, Fisher’s exact test). The DNA yield was increased in the AgST compared with the ST and SCF samples, particularly for PL, VF, AF, and PF, with very low microbial loads (Fig. 1b).

We then evaluated microbial DNA in the samples using quantitative PCR (qPCR) of the 16S rRNA gene V1–V2 region. More microbial DNA was found in the AgST than in the other solutions for all skin sites. Values significantly differed between AgST and SCF samples for PL, AF, and PF and between AgST and ST samples for VF, AF, and CH (Fig. 1c). In addition, the AgST samples contained more 16S rRNA gene abundance than the ST and SCF samples in 60 and 57 of the 66 samples, respectively. Three FH samples with relatively high microbial loads did not differ significantly (Fig. 1c).

### Effects of increased microbial DNA on the skin microbiome structure

**Figure 2:**
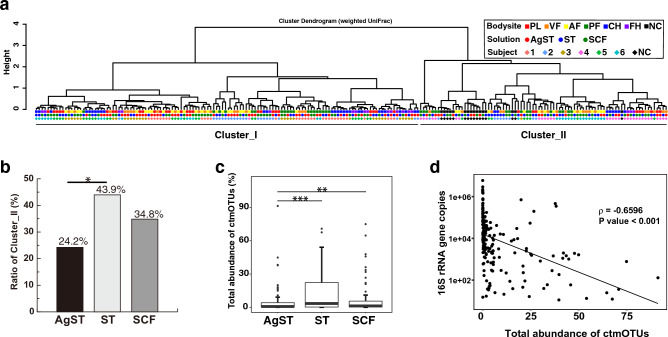
Effect of agar-containing sampling solutions on skin microbiome structures. (**a**) A cluster dendrogram was created based on hierarchical clustering of weighted UniFrac distance between all 198 samples and 16 negative control (NC) samples. These samples were divided into 2 clusters: Cluster I contained 130 skin samples and Cluster II contained 68 skin samples and 16 NC samples. (**b**) The ratio of skin samples divided into Cluster II, including NC samples is shown in the bar plot. The ratios of AgST, ST, and SCF samples were 24.2%, 43.9%, and 34.8%, respectively. AgST and ST differed significantly from each other. * P < 0.05 (Fisher’s exact test). (**c**) Relative abundance of ctmOTUs among the three sampling solutions. * *P* < 0.05 and *** *P* < 0.001 (Wilcoxon signed-rank test). (**d**) Scatter plot shows moderate negative correlation between 16S rRNA gene copies and total relative abundance of ctmOTUs (ρ = -0.6596, *P* < 0.001; Spearman’s rank correlation test).

We evaluated the effect of increased microbial DNA recovered in AgST on the structure of the skin microbiome. We clustered high-quality 16S rRNA gene amplicon reads from 198 skin samples with 97% identity to construct the operational taxonomic unit (OTU) and obtained 3699 OTUs. The 16S rRNA gene amplicon sequencing data derived from 198 skin samples and 16 sampling solutions without swabbing skin (negative controls; NCs) on different days were hierarchically clustered based on weighted UniFrac distances (Fig. 2a). Cluster I comprised skin samples only (N = 130), while cluster II comprised skin samples (N = 68) and NCs (N = 16). Cluster II contained 16 (24.2%), 29 (43.9%), and 23 (34.8%) skin samples with AgST, ST, and SCF, respectively. (Fig. 2a). Cluster II contained significantly fewer AgST samples than ST samples (*P* = 0.02701, Fisher’s exact test) (Fig. 2b). Comparisons of 16S rRNA gene copy numbers in samples revealed significantly more microbial DNA in Cluster I than in Cluster II (Supplementary Fig. S1). Although Cluster II contained most of the samples from participants 4 and 6, AgST samples from participant 6 belonged to Cluster I due to an increased amount of microbial DNA. These data demonstrate that the quantity of microbial DNA in skin microbiome samples affected clustering.

We investigated the microbial composition of all skin samples and NCs at the OTU level. A MaAsLin2 comparison of 130 skin samples (without 68 skin samples in Cluster II) and 16 NCs revealed that 152 of 3,699 OTUs were enriched in NCs. These were defined as contamination-related (ctmOTUs; coefficient > 0, *P* < 0.05). Of the 152 ctmOTUs, 70 were specific to NCs. The median ctmOTU abundance in NCs was 90.9%, which was significantly higher than the samples classified in Clusters I and II (median, 0.4% and 15.3%, respectively) (Supplementary Fig. S2).

The 152 ctmOTUs were assigned to 10 phyla, including 83 *Proteobacteria* (54.6%), 29 *Firmicutes* (19.1%), 20 *Bacteroidetes* (13.2%), 12 *Actinobacteria* (7.9%) species and six other phyla (5.3%). Of the 152 OTUs, 94 (belonging to 49 genera) were previously reported as contaminants in the works of Salter et al.^11^ and Marsh et al.^16^, as well as in the RIDE checklist^22^ (Supplementary Table S2). The total abundance of ctmOTUs in the AgST samples was significantly lower than in SCF and ST samples (*P* = 0.0063 and *P* = 0.0001, respectively; Fig. 2c). The total ctmOTU abundance and 16S rRNA gene copy numbers were negatively correlated (ρ = −0.6596 and *P* = 4.27e-26, respectively; Spearman rank correlation tests; Fig. 2d).

### Contribution of agar to improving microbial DNA recovery

**Figure 3:**
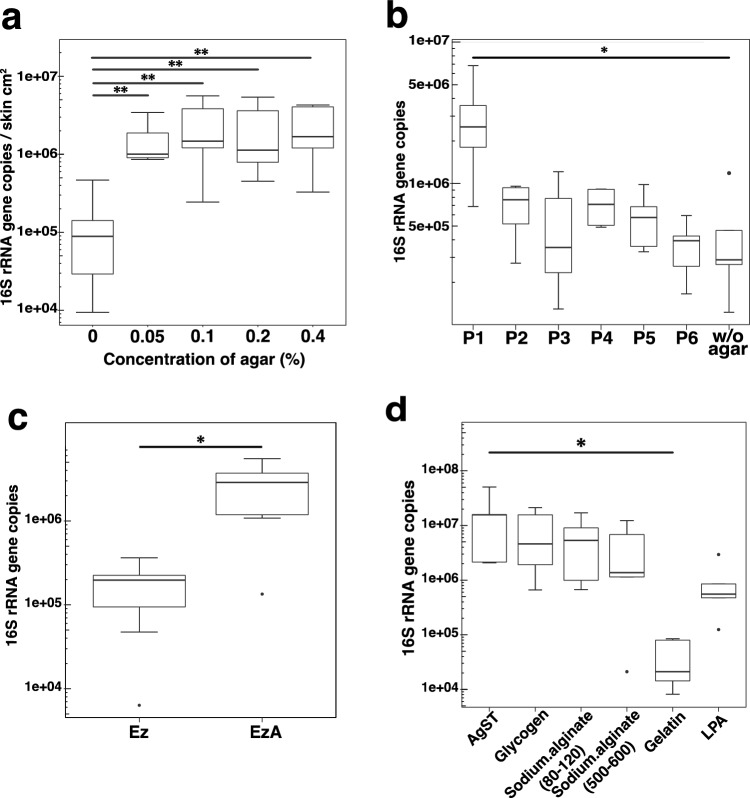
Contribution of agar under different DNA extraction conditions. (**a**) Boxplots show comparisons of 16S rRNA gene copies per cm^2^ of skin from DNA extracted by enzymatic lysis with five agar concentrations. ** *P* < 0.05, 0% vs. 0.05%, 0.1%, 0.2%, or 0.4% (w/v) agar (Steel’s multiple comparison (two-sided) test). (**b**) P1–6 indicate the point at which agar was added to the solutions. Boxplots show 16S rRNA gene copies during each enzymatic lysis process. The 16S rRNA gene copies on P1 were highest within all processes. Without (w/o) agar indicates 16S rRNA gene copies in the absence of agar during the entire DNA extraction. * *P* < 0.05 vs. control w/o agar (Steel’s multiple comparison (one-sided) test). (**c**) Boxplots show the comparisons of 16S rRNA gene copies between the enzymatic lysis method with agar without bacterial cell precipitation (EzA) and the enzymatic lysis method without agar and bacterial cell precipitation (Ez). * *P* < 0.05 (Wilcoxon signed-rank test). (**d**) Boxplot showing 16S rRNA gene copies with AgST and the other co-precipitants after enzymatic lysis. Co-precipitants comprised 0.2% (v/v) glycogen, 0.2% (w/v) sodium alginate (80–120), 0.2% (w/v) sodium alginate (500–600), 0.2% (w/v) gelatin, and 0.2% (v/v) LPA. * *P* < 0.05 (Steel’s multiple comparison (two-sided) test with AgST as the control).

We assessed the optimal agar concentration required in the sample solution to maximize DNA recovery using forehead skin samples obtained from seven volunteers. The microbial DNA yield was similar in solutions containing 0.05‒0.4% (w/v) agar (Fig. 3a). We then investigated the experimental steps involved in the agar-enhanced recovery of community DNA. We evaluated the swabbing skin process according to the sampling solutions. The 16S rRNA gene copy number did not significantly differ between skin samples collected with AgST and skin samples collected with ST and supplemented with agar before DNA extraction (ST + agar) (Supplementary Fig. S3). This finding suggests that the heightened amount of microbial DNA could not be attributed to the presence of agar during skin swabbing. Instead, it was due to the presence of agar in the reaction solution at the beginning of the DNA extraction process.

We added 0.2% agar (w/v) at six specific points during the DNA extraction process of saliva samples diluted 1,000-fold, which contained microbial loads comparable to those of skin samples. The six points were labeled as follows: P1 before bacterial cell precipitation, P2 before enzymatic lysis, P3 before phenol–chloroform extraction, P4 before isopropanol precipitation, P5 before RNase treatment, and P6 before polyethylene glycol precipitation (Supplementary Fig. S4). We then compared the microbial DNA yield using qPCR of the 16S rRNA gene (Fig. 3b). As the timing of agar addition during DNA extraction became later, the DNA yield tended to decrease. Adding agar at P1 resulted in significantly higher DNA generation compared to the absence (w/o) of agar (*P* = 0.038; Steel’s multiple comparison; Fig. 3b).

Significantly more microbial DNA was obtained via enzymatic lysis with agar and without centrifugation compared to without both agar and centrifugation (*P* = 0.0156; Wilcoxon signed-rank test) (Fig. 3c). These data suggest that agar functions as a co-precipitant during the precipitation of microbial cells and DNA. Therefore, we also tested other co-precipitants, including glycogen, sodium alginate, gelatin, and linear polyacrylamide (LPA). Glycogen and sodium alginate similarly increased the DNA yield to that of AgST, whereas gelatin and LPA did not (Fig. 3d).

### Bacterial DNA yield from low-biomass samples was higher using enzymatic lysis with agar than conventional methods

We compared microbial DNA yield from 1000-fold diluted saliva between enzymatic lysis with and without agar, and two commercially available kits: PowerSoil DNA Isolation Kit (MB) and PureLink Genomic DNA Mini Kit (PuLi). We extracted DNA from saliva without precipitating microbial cells.

**Figure 4:**
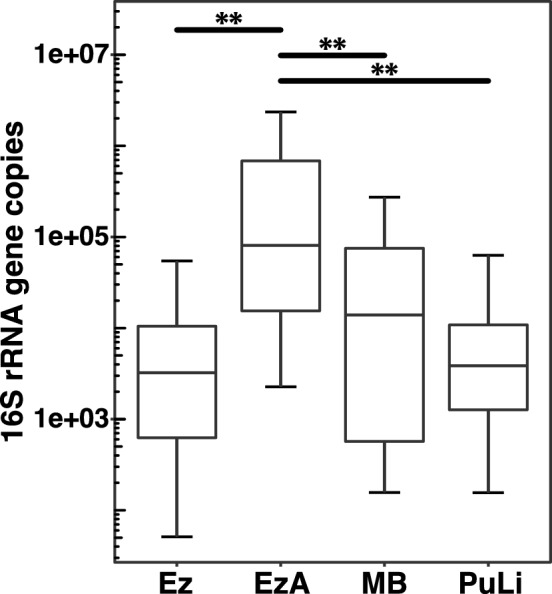
Comparison of 16S rRNA gene copies under four different conditions of DNA extraction. Boxplots show comparisons of 16S rRNA gene copies under four DNA extraction conditions. Ez, enzymatic lysis; EzA, enzymatic lysis with agar; MB, PowerSoil DNA Isolation Kit; PuLi, PureLink Genomic DNA Mini Kit. ** *P* < 0.01 (Wilcoxon signed-rank test).

The contribution of agar varied depending on the method used. Microbial DNA yields were significantly better when agar was included in enzymatic lysis compared with MB and PuLi. Microbial DNA yield using enzymatic lysis tended to decrease the DNA yield compared with the other methods. Adding agar to the MB significantly increased the microbial DNA yield, but it remained significantly lower than that generated by enzymatic lysis with agar (Fig. 4, Supplementary Fig. S5).

## Discussion

Recently, there has been increasing recognition of the significance of the microbiome at low-biomass sites. For example, microbiome analysis at certain low-biomass sites, including the skin, lungs, and female reproductive organs, has revealed their importance^23–25^. The specificity of the skin microbiome has been determined, and it has already been included in forensics investigations and its application in criminal investigations is also being discussed ^26–28^. However, these findings are limited, and little is known about the microbiomes of low-biomass sites due to analytical challenges^10^. To accurately understand the structure of these microbiomes and verify their biological significance, DNA loss during the experimental process must be reduced to ensure a sufficient amount of microbial DNA input for accurate analysis. Explanations for why the analysis of microbiomes at low-biomass sites is so difficult include the low abundance of microbial DNA extracted from samples and contamination effects. Appropriate negative control samples have been established to assess contamination effects, and benchmarks of commercially available kits have been evaluated^12,15,16^. However, a practical method has yet to be proposed. The present study aimed to increase microbial DNA recovery by improving current extraction methods using simple materials based on detailed verification. We showed that agar addition during DNA extraction greatly improved microbial DNA yields and mitigated the effects of contamination, thereby enabling the analysis of a greater number of samples.

We evaluated the influence of agar on experimental processes to understand its roles during DNA extraction procedures. Regardless of whether the solution contained agar during skin swabbing, the 16S rRNA gene copy number was maximized when agar was added during DNA extraction. This result suggests that agar functions as a co-precipitant of DNA and microbial cells and reduces DNA loss during the entire experimental process. Agar is more useful than other DNA co-precipitants because it is practical, inexpensive, and maximizes DNA recovery when added during enzymatic lysis. Agar also improved other DNA extraction methods, but not to the same degree as enzymatic lysis. Furthermore, the simplicity of agar addition could be easily integrated into automated workflows. In recent years, the introduction of automated devices has been advancing to streamline the analysis of microbiomes and to process a large number of samples. It would be important to explore the application of this method to existing automated workflows in the future. Compared with commercially available kits, enzymatic lysis requires several experimental steps that can cause DNA loss, including DNA precipitation and supernatant collection. The utility of enzymatic lysis-based methods for DNA extraction from low-biomass sites have been underestimated, as more DNA is lost compared with the other methods. However, compared to popular kits, enzymatic lysis with agar significantly increased DNA yields; thus, this method appears to be the most effective approach for analyzing low-biomass samples. Enzymatic lysis with agar can help prevent the loss of valuable information from samples and enhance the potential of microbiome analysis at low-biomass sites. Most studies of such microbiomes have been performed using 16S rRNA gene amplicon sequencing. However, enzymatic lysis with agar may also facilitate metagenomic analysis. Previously, the enzymatic lysis method improved the recovery of high-molecular-weight DNA and was advantageous for long-read metagenomic sequencing^29^. Therefore, our method of combining AgST with enzymatic lysis might improve the throughput and read length of long-read metagenomics of the skin microbiome. Additionally, future research could explore the potential impact of enzymatic agar methods on DNA quality in skin samples. Although long-read metagenome sequencing of the skin microbiome remains challenging, enzymatic lysis with agar might help overcome this challenge.

We also defined ctmOTUs based on the hierarchical clustering of each sample. Although previous reports have identified candidate bacterial species related to contamination, we doubt the relevance of establishing a consistent relationship between specific species and contamination^11,14,16,22^. For example, *Cutibacterium* gen. species are abundant in skin samples and are often treated as contaminants in saliva analysis but not in skin analysis. Thus, we believe that analyzing the microbiome at low-biomass sites requires the establishment of contamination thresholds based on data-driven determinations for each analysis. The thresholds determined in this study may not be directly applicable to others, but we hope that they can be applied in future analyses as references. Furthermore, our results cannot be interpreted as a universal increase in DNA yield, as our assessment of the solutions was conducted at one time point for each sample.

## Conclusion

We developed a method to increase the yield of microbial DNA during enzymatic DNA extraction by adding agar to the reaction solution. The most important aspect of this method is the addition of agar to the reaction solution at a point before enzymatic lysis begins. Moreover, this increase was similar for agar concentrations of 0.05–0.4%. This suggests that there is some flexibility in the addition of agar, and our method may be applicable to different sample collection protocols and storage conditions. The combination of enzymatic lysis with agar significantly improved DNA extraction from low-biomass samples and enabled microbiome analysis without contamination. Our method should facilitate advanced studies, such as metagenomic analysis of low-biomass samples, which have been technically hampered by the need for large amounts of DNA. Based on the above, we strongly recommend using enzymatic lysis with agar to extract DNA from low-biomass samples. We also defined a standard for determining contaminating species. We believe that this threshold could be helpful for future studies.

## Materials and methods

### Ethical considerations

This study complies with relevant institutional, national, and international guidelines and legislation. The Ethics Committee at the RIKEN Center for Integrative Medical Sciences (H30-4) approved this study, and written informed consent was obtained from all volunteers who provided specimens.

### Sampling solutions

The AgST solution was prepared as follows: 0.15 M sodium chloride containing Tween-20 (0.1% v/v) and agar (0.05%, 0.1%, 0.2%, or 0.4% w/v) was sterilized by autoclave at 121 °C for 30 min using Saniclave-102 (Revolutionary Science, Shafer, MN, USA). and then cooled with stirring to 22–24 °C. AgST with 0.2% (w/v) agar was utilized for all experiments except for the agar concentration assessment experiment. The ST solution, consisting of 0.15 M sodium chloride and 0.1% Tween-20 and the SCF solution, comprising 50 mM Tris–HCl, 1 mM EDTA and Tween-20 (0.5% v/v), were sterilized by filtration through a membrane with 0.22-µm pores. Before using the prepared reagents, we assessed the level of contamination by conducting qPCR on the solution in its original state and after undergoing the DNA extraction process.

### Sample collection

Skin samples were collected over a period of 3 to 14 days from six healthy Japanese volunteers more than 4 h after washing and without applying any skincare products. Skin samples were collected from 40 cm^2^ areas of both sides of the palm (PL), volar forearm (VF), antecubital fossa (AF), popliteal fossa (PF), and forehead (FH), as well as a 20 cm^2^ area of the cheek (CH), yielding a total of 11 sampling sites. Samples were collected using MENTIP sterile cotton-tipped swabs (JCB Industry Ltd., Tokyo, Japan) moistened with each of the solutions described above. The swabs were resuspended manually in a 1.5 mL solution of AgST, ST, or SCF to release microbes. Skin samples were immediately frozen in liquid nitrogen and stored at −80 °C.

We collected 2 mL saliva samples from 11 healthy Japanese volunteers at random times during the day, excluding a 2-h period after meals. The samples were diluted 100-fold in phosphate-buffered saline (Thermo Fisher Scientific Inc., Waltham, MA, USA), flash-frozen in liquid nitrogen, and stored at −80 °C. Samples were further diluted tenfold for DNA extraction. To collect the NCs, sterile cotton-tipped swabs immersed in the three sampling solutions (without skin samples) were exposed to the air in the experimental environment. Skin samples and NCs were collected on different days to avoid cross-contamination.

### Test sample preparation for comparing DNA co-precipitants

We used five reagents known as DNA co-precipitants: glycogen (Fujifilm Wako Pure Chemical Corp., Osaka, Japan), sodium alginate with viscosity range 80–120 mPa·s (10 g/L, 20℃; Fujifilm Wako Pure Chemical Corp.), sodium alginate with viscosity range 500–600 mPa·s (10 g/L, 20℃; Fujifilm Wako Pure Chemical Corp.), gelatin (Fujifilm Wako Pure Chemical Corp.), and LPA (Ethachinmate, Nippon Gene Co., Ltd., Tokyo, Japan) These reagents were added to 1,000-fold diluted saliva at a final concentration of 0.2%.

### DNA extraction and quantitation

To minimize the risk of contamination, we performed the experiments in a PCR workstation (UV3 HEPA PCR workstation, UVP, Germany) specifically during the processes of DNA extraction, PCR reaction, and purification of PCR products. Microbial DNA was prepared using enzymatic lysis^21^ with modifications. Frozen samples were thawed on ice and then centrifuged for 15 min at 12,000 × g at 4 °C. Pellets were resuspended in 10 mM Tris and 20 mM EDTA (TE20). Microbial cells were enzymatically lysed using 7.5 mg of lysozyme (Sigma-Aldrich Co., St. Louis, MO, USA) and 1,000 units of purified achromopeptidase (Fujifilm Wako Pure Chemical Corp.) at 37 °C for 2 h, followed by a final concentration of 1% sodium dodecyl sulfate (Fujifilm Wako Pure Chemical Corp.) and 0.5 mg proteinase K (Merck KGaA., Darmstadt, Germany) at 55 °C for 1 h. Lysates were then gently mixed for 10 min at 24℃ in equal volumes of phenol, chloroform, and isoamyl alcohol (Nippon Gene Co. Ltd.) in a ratio of 25:24:1. We added 3 M sodium acetate (10% v/v) to the supernatants collected by centrifugation. Thereafter, DNA was precipitated in isopropanol, pelleted by centrifugation at 15,000 × g for 15 min at 4 °C, rinsed once with 75% ethanol, and dissolved in 100 µL of 10 mM Tris and 1 mM EDTA (TE) buffer. The DNA was treated with 10 mg/mL RNase A (Nippon Gene Co. Ltd.) for 30 min at 37 °C, mixed gently with 0.6 volumes of 20% polyethylene glycol 6,000 and 2.5 M sodium chloride (Hampton Research Corp., Aliso Viejo, USA), and placed on ice for at least 10 min. The DNA was pelleted by centrifugation at 15,000 × g for 15 min at 4 °C, rinsed twice with 75% ethanol, and dissolved in TE buffer (50 µL).

The total DNA yield was measured using Qubit dsDNA HS Assay Kits and a Qubit 3.0 or 4.0 fluorometer (Thermo Fisher Scientific Inc.). Microbial DNA in each sample was quantified using the ABI StepOnePlus real-time quantitative PCR system (Thermo Fisher Scientific Inc.) in FAST mode and universal primers for the 16S rRNA gene V1–V2 region 27Fmod (5ʹ-agrgttgatymtggctcag-3ʹ) and 338R (5ʹ-tgctgcctcgtaggagt-3ʹ). The PCR reaction mix comprised 2 µL of DNA template, 10 µL of Fast SYBR Green Master Mix (Thermo Fisher Scientific Inc.), 20 pmol each of the forward and reverse primers, and brought to a total volume of 20 µL with DNA-free water. qPCR was performed with the following cycling conditions: 95 °C for 20 s, followed by 40 cycles of 95 °C for 3 s and 60 °C for 30 s. The *Escherichia coli* 16S rRNA gene sequence was used as a reference for the generation of standard curves. The standard curve utilized a series of tenfold serial dilutions ranging from 0.1 ng/µL to 1.0 × 10^–7^ ng/µL, resulting in seven levels of diluted standards.

### 16S rRNA gene amplicon sequencing and data processing

The 16S rRNA gene V1–V2 region was amplified using a 9,700 PCR System (Life Technologies, Tokyo, Japan) with a unique 8-bp index (indicated by NNNNNNNN) attached to both the forward 27Fmod^30^ (5ʹ-AATGATACGGCGACCACCGAGATCTACAC NNNNNNNN ACACTCTTTCCCTACACGACGCTCTTCCGATCT agrgtttgatymtggctcag-3ʹ) and reverse 338R (5ʹ-CAAGCAGAAGACGGCATACGAGAT NNNNNNNN GTGACTGGAGTTCAGACGTGTGCTCTTCCGATCT tgctgcctcccgtaggagt-3ʹ) primers. The PCR reaction mix comprised 4 µL of DNA template, 0.5 µL of ExTaq polymerase (Takara Bio Inc., Kusatsu, Japan), 5 µL of ExTaq buffer, 5 µL of dNTPs, 10 pmol each of the forward and reverse primers, and brought to a total volume of 50 µL with DNA-free water. Amplicons generated under 30 cycles of 98℃ for 30 s, 55℃ for 45 s, and 72℃ for 2 min were purified using AMPure XP (Beckman Coulter Inc., Brea, CA, USA). The amplicons were pooled at the same concentration to generate a multiplexed MiSeq library. Sequencing was performed using the MiSeq sequencing system (Illumina Inc.), as described by the manufacturer, with MiSeq Reagent Kit v3 (600 cycles).

Raw reads generated by MiSeq were processed in our in-house pipeline, as described previously^31^, with slight modifications. Briefly, paired reads were joined using fastq-join v1.3.1 (https://github.com/brwnj/fastq-join). The reads that were not detected with primer sequences at both ends were removed using a BLAST search. The primer sequences were then trimmed from the remaining reads. Primer-trimmed reads with an average quality value < 25 were removed. Reads that had the highest similarity to eukaryotes and hit the bacterial genome database with < 90% coverage were further filtered out. The remaining reads were regarded as high quality, and we randomly selected 3,000 of these reads per sample. The Good’s coverage index of 3,000 reads per sample exceeded 0.984, indicating a high degree of coverage, which was sufficient for microbiome analysis^32,33^. The selected reads of all samples were first sorted by the frequency of redundant sequences and grouped into OTUs using UCLUST, with a sequence identity threshold of 97%. Sequences with the highest redundancy in each OTU were determined as representative. We used GLSEARCH to search for homology between representative OTU sequences and the 16S database in order to determine their classification. We assigned sequence similarity thresholds of 70%, 94%, and 97% at the phylum, genus, and species levels, respectively. The 16S rRNA gene database was reconstructed and curated from the following publicly available sources: Ribosomal Database Project (RDP; Release 11, Update 5: September 30, 2016), CORE (http://microbiome.osu.edu/, updated October 13th, 2017), genomic-based 16S rRNA gene database (GRD; updated April 2013), and RefSeq 16S rRNA gene database (downloaded May 7, 2019, from NCBI).

Beta diversity was evaluated by computing weighted and unweighted UniFrac distances between the samples. Hierarchical clustering was analyzed as described by Ward^34,35^.

### Statistical analysis

All data were statistically analyzed using R. Microbiome data were compared between groups using Wilcoxon rank-sum and signed-rank tests for two-group comparisons, and the Benjamini–Hochberg procedure was applied for comparisons involving three or more groups. Microbial DNA concentrations were compared between the controls and other groups using Steel’s multiple comparison tests. Nonrandom associations between pairs of categorical variables were assessed by Fisher’s exact tests using the *fisher.multicomp* function of the *RVAideMemoire* package (https://cran.r-project.org). The significance level was set at *P* < 0.05 throughout this study. MaAsLin2 was run using the *Maaslin2* package with min_prevalence > 0 and all other default settings.

### Supplementary Information

Supplementary Information 1.

Supplementary Information 2.

## Data Availability

The 16S rRNA gene V1–V2 region sequence data generated and/or analyzed during the current study are available in the DDBJ repository, PRJDB15042: https://ddbj.nig.ac.jp/resource/bioproject/PRJDB15042.
