# Supplementary material for: Recovery of microbial DNA by agar-containing solution from extremely low-biomass specimens including skin
Source: Sci Rep. 2023 Nov 11;13:19666. doi: 10.1038/s41598-023-46890-7 (PMC10640576; doi:10.1038/s41598-023-46890-7)
Supplement: Supplementary file 1 — Supplementary Information 1. [file 41598_2023_46890_MOESM1_ESM.pdf]

## Supplementary Information

**Title**

Recovery of microbial DNA by agar-containing solution from extremely low-biomass specimens including skin

**Authors**

Rina Kurokawa, Hiroaki Masuoka, Lena Takayasu, Yuya Kiguchi, Yusuke Ogata, Ryoko Miura-Kawatsu, Masahira Hattori, Wataru Suda

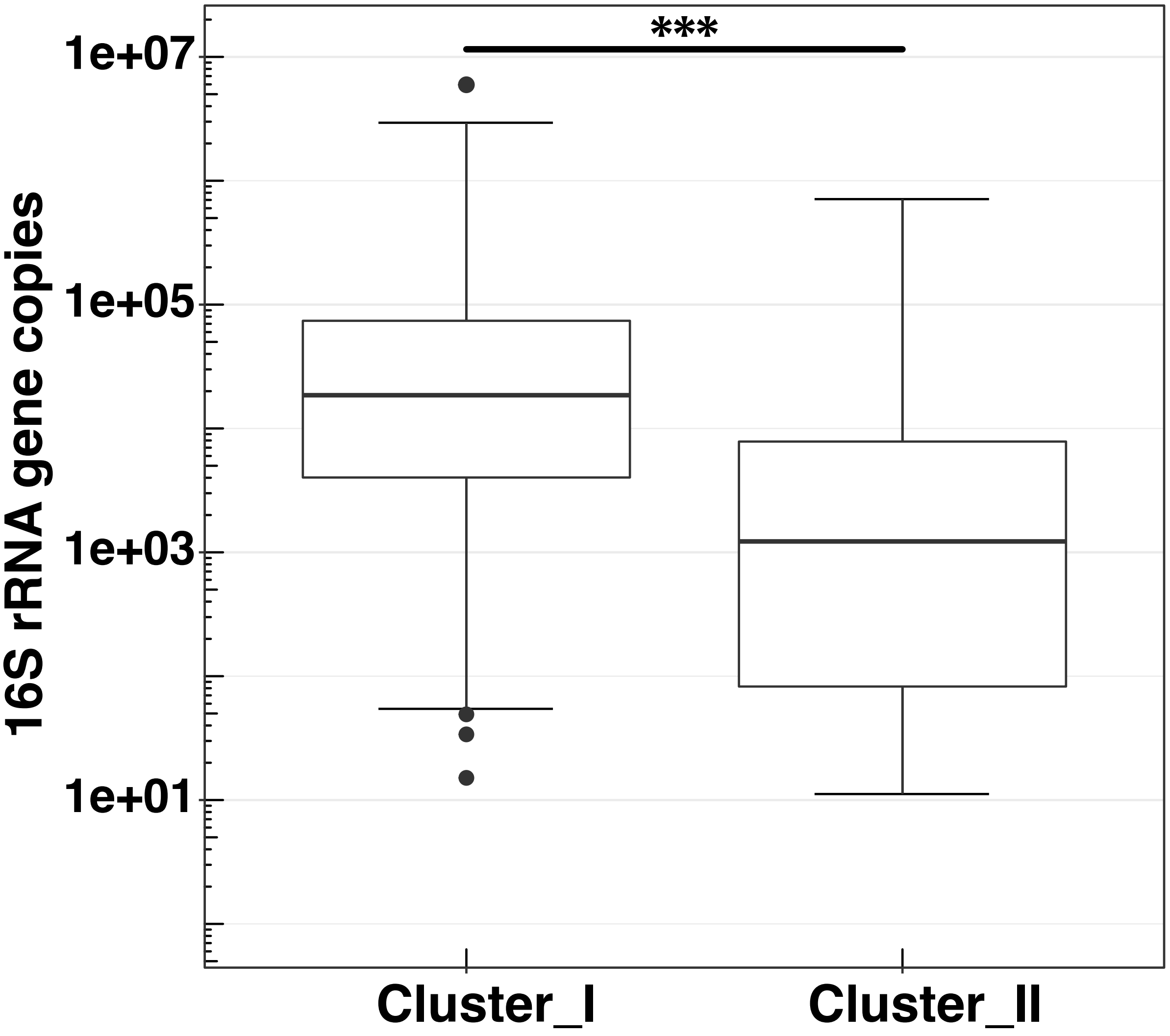


**Supplementary Figure S1: Boxplots for comparison of 16S rRNA gene copy numbers in Clusters I and II.**

Vertical axis indicates the number of 16S rRNA gene copies per $cm^2$ of skin samples swabbed using three sampling solutions. *** $P < 0.001$ (Wilcoxon rank-sum test).

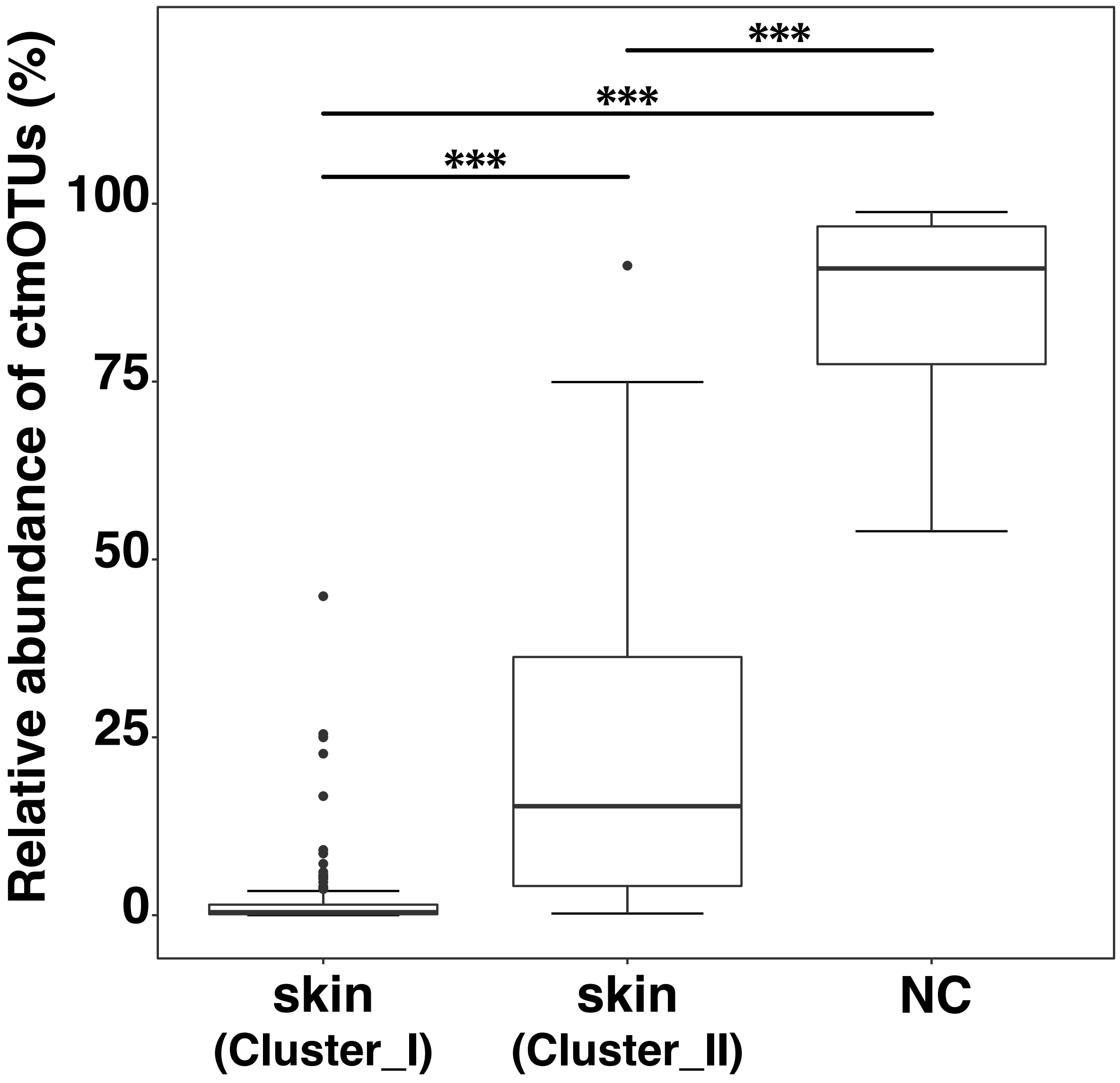


**Supplementary Figure S2: Total abundance of ctmOTUs in skin and NC samples.**

Boxplot shows relative abundance of 152 ctmOTUs in skin samples and NC samples. *** $P < 0.001$ (Wilcoxon rank-sum test). ctmOTUs, contamination-related operational taxonomic units; NC, negative control.

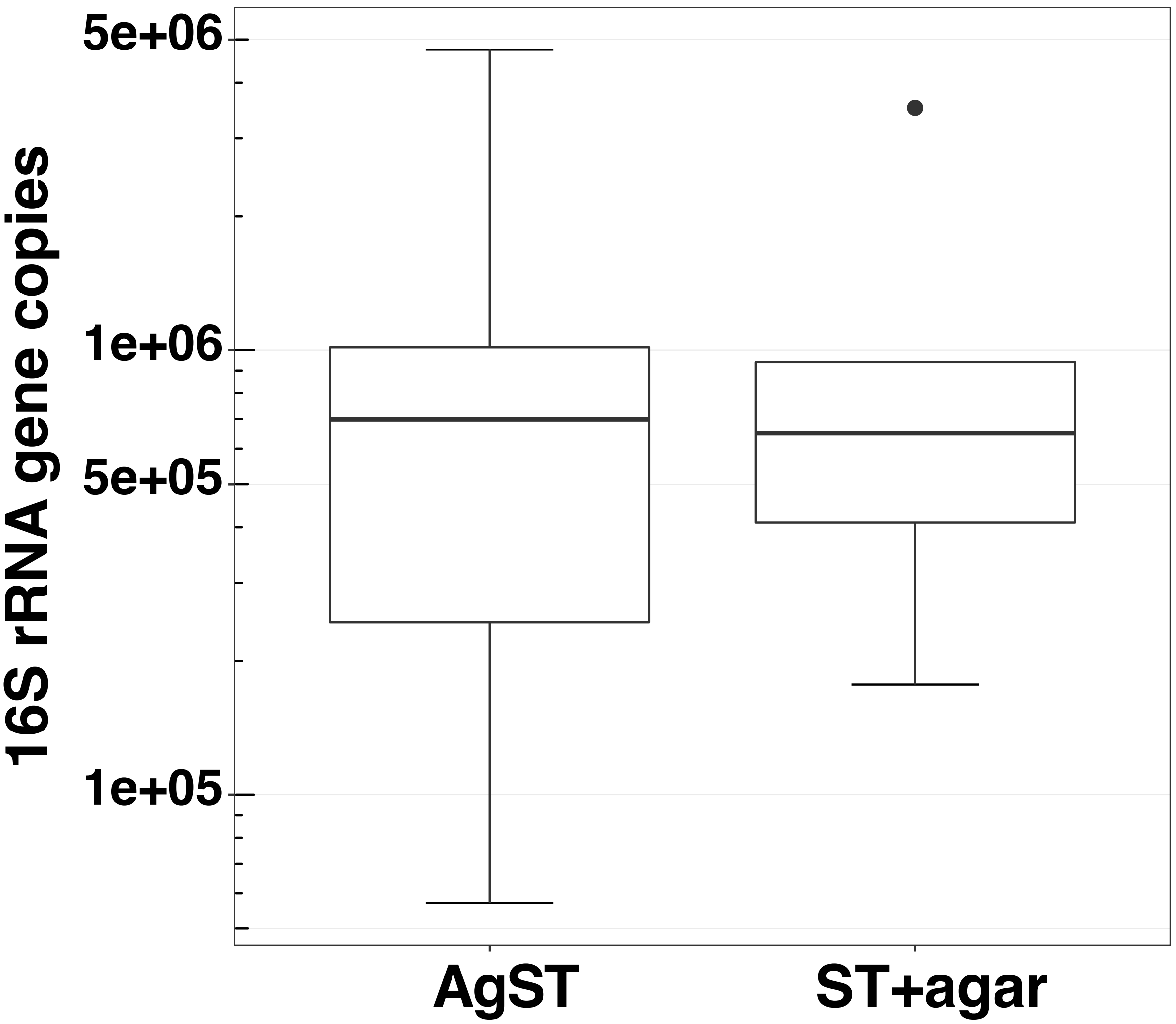


**Supplementary Figure S3: Comparison of the 16S rRNA gene copy numbers between samples swabbed with AgST or ST+agar.**

Boxplots show comparisons of 16S rRNA gene copies. ST+agar indicates the skin samples swabbed with ST solution and supplemented with agar before DNA extraction. No significant difference was found between AgST and ST+agar (Wilcoxon signed-rank test).

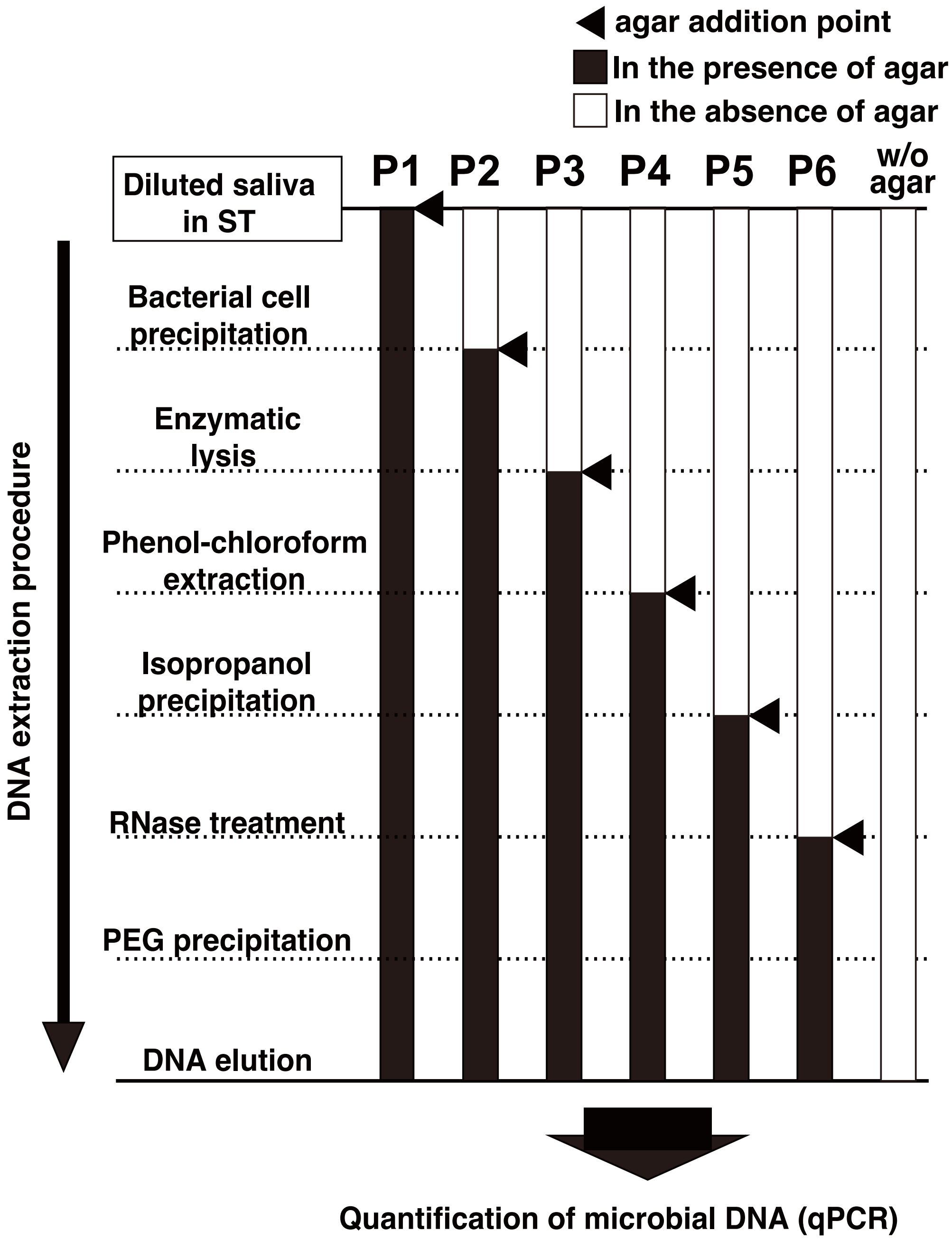


**Supplementary Figure S4: The process of determining the optimal step for adding agar in the enzymatic DNA extraction method.**

The vertical axis indicates the enzymatic lysis DNA extraction procedure, which was performed from the top down. P1–6 and w/o agar indicate diluted saliva samples with agar addition at different time points; w/o, without. Black triangles indicate the timing of agar addition, which was before the procedure below each triangle. The black bars represent the DNA extraction procedure in the presence of agar, while the white bars indicate the DNA extraction procedure in the absence of agar. All samples underwent the complete DNA extraction process, followed by quantification of microbial DNA using qPCR. The results are shown in Figure 3b.

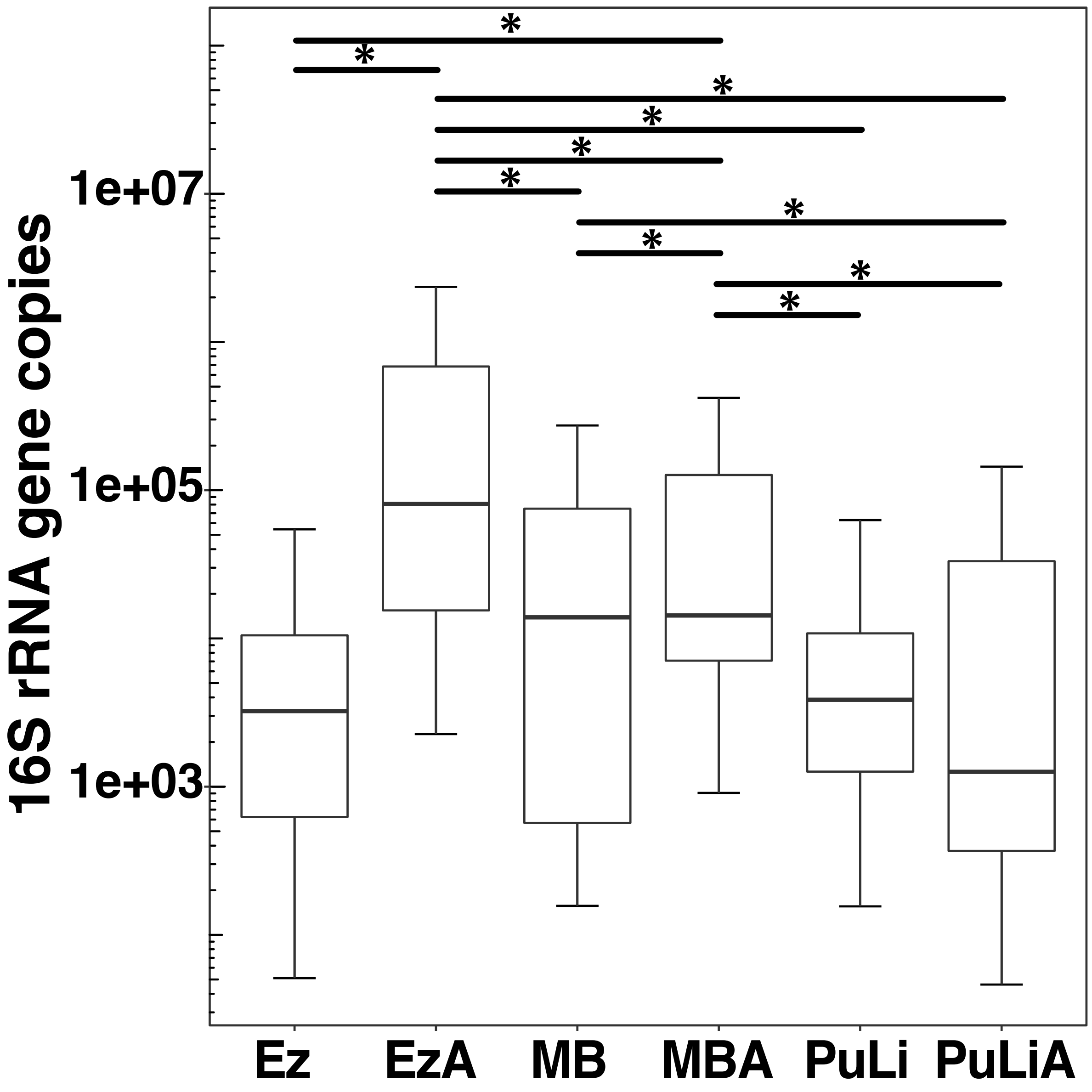


**Supplementary Figure S5: Contribution of agar to different DNA extraction methods.**

Boxplots show comparisons of 16S rRNA gene copies obtained under six DNA extraction conditions: Ez, Enzymatic lysis; EzA, Enzymatic lysis with agar; MB, PowerSoil DNA Isolation Kit; MBA, PowerSoil DNA Isolation Kit with agar; PuLi, PureLink Genomic DNA Mini Kit; PuLiA, PureLink Genomic DNA Mini Kit with agar. *$P < 0.05$ (Wilcoxon signed-rank test).
